# TALEN-based editing of *TFIIAy5* changes rice response to *Xanthomonas oryzae* pv. *Oryzae*

**DOI:** 10.1038/s41598-020-59052-w

**Published:** 2020-02-06

**Authors:** Jin Han, Zhihui Xia, Pengcheng Liu, Chunrong Li, Yanyan Wang, Lequn Guo, Guanghuai Jiang, Wenxue Zhai

**Affiliations:** 1 Institute of Genetics and Developmental Biology, Chinese Academy of Sciences, Beijing, 100101 China; 2 Institute of Tropical Agriculture and Forestry, Hainan University, Haikou, 570228 China

**Keywords:** TAL effector nuclease, Molecular engineering in plants

## Abstract

The *xa5* gene encodes a basal transcription factor (TFIIAγ) protein with wide spectrum resistance to bacterial blight caused by *Xanthomonas oryzae pv. Oryzae* (*Xoo*) in rice. It was only found in a few rice ecotypes, and the recessive characteristics limited its application in breeding. Here, we employed a TALEN-based technique to edit its dominant allelic *TFIIAγ5* and obtained many mutant *TFIIAγ5* genes. Most of them reduced rice susceptibility to varying degrees when the plants were challenged with the *Xoo*. In particular, the knocked-out *TFIIAγ5* can reduce the rice susceptibility significantly, although it cannot reach the *xa5-*mediated resistance level, indicating *TFIIAγ5* is a major component involved in disease susceptibility. In addition, the mutant encoding the protein with deletion of the 32nd amino acid or amino acid insertion between 32nd and 33rd site confers rice with the similar resistance to that of the knocked-out *TFIIAγ5*. Thus, the amino acids around 32nd site are also the important action sites of TFIIAγ5 besides the 39th amino acid previously reported. Moreover, the integration of *xa5* into *TFIIAγ5*-knockout plants conferred them with a similar resistance as IRBB5, the rice variety containing the homozygous *xa5* gene. Thus, *TFIIAγ5* was not simply regarded as a resistant or a susceptible locus, as the substitution of amino acids might shift its functions.

## Introduction

Plant diseases caused by the bacterial from genus *Xanthomonas* can reduce the yield and quality in a variety of crops. During infection, the *Xanthomonas* bacterium delivers types of effectors into host cells through a type III secretion (T3S) pathway. Transcription activator-like effectors (TALEs) are major virulence factors in *Xanthomonas*. They modulate host transcription to facilitate pathogen growth or propagation^1^. TALEs can trans-activate host genes by directly binding to their effector binding elements (EBEs) in the DNA^2^. In addition, TALEs can combine other protein factors to control gene expression or to alert protein function, besides binding to DNA directly. Some truncated TALEs, also termed interfering TALEs or iTALEs, were used by the pathogen *Xanthomonas oryzae* and suppressed the disease resistance of plants by altering the action of resistant factors^3,4^. However, some TALEs such as AvrXa10^5^, AvrXa27^6^, and AvrXa23^7^ can activate resistant genes in plants.

Bacterial blight caused by *Xanthomonas oryzae* pv. oryzae (*Xoo*) is a well-studied disease in rice. In the interaction between *Xoo* and rice, the TALEs were shown to play important roles in regulating the expressions of rice susceptible or resistant genes. To date, more than 40 bacterial blight resistant (R) genes in rice have been identified^8^ and eleven of them have been cloned and characterized^5–7,9–16^. Among these cloned genes, the dominant R genes *Xa1*, *Xa10*, *Xa23*, and *Xa27* can be induced by TALEs to trigger hypersensitive response (HR) and cell death^3,5–7^. The recessive R genes such as *xa13*, *xa25*, and *xa41* confer rice resistance because the TAL effectors cannot recognize their promoter to activate them^16–19^. However, the recessive R gene *xa5* expresses constitutively and encodes a TFIIA*γ*-like protein, indicating that it behaves differently from other R genes in the *Xoo*-rice interaction.

TFIIA*γ* is a general transcription factor involved in RNA polymerase II-dependent transcription in higher eukaryotes^20^. In rice, the OsTFIIA*γ5/Xa5* gene is showed to play an important role response to bacterial pathogens^21^. Xa5/TFIIAγ5 can work as a key component in the disease resistance mediated by some dominant R genes, such as *Xa27*^22^ and *Xa10*^5^. On the other hand, it was shown to interact directly with TALEs to activate disease susceptibility genes. The attenuation or block of this interaction could eliminate or weaken the activation of rice susceptibility gene expression^21^. The two-nucleotide change in its second exon, resulting in the produce of recessive gene *xa5*, encoding a protein with the substitution of glutamic acid at 39th position to valine (E39V)^13,23^. The *xa5* gene is also very important in rice breeding for its broad resistance spectrum to most *Xoo* strains, except a few strains employing the TALE of pthXo1^24^. The pyramid lines of the *xa5* gene and some other dominant genes such as *Xa21* and *Xa4* have a higher and wider spectrum resistance than the plants harboring only one BB resistance gene^25^. Nevertheless, the *xa5* is a recessive gene and found only in a few rice ecotypes^26^, restricting its application on rice breeding. These previous studies may provide us some new ideas to use Xa5/TFIIAγ5 genes for breeding purposes.

Transcription activator-like effector nucleases (TALENs) are programmable nucleases in which a truncated TALE is linked to the catalytic domain of FokI for targeted genetic modifications. The target recognition is commanded by assembling TALEs using a code-like modularity of TALE-DNA recognition^27^. TALEs possess an N-terminal T3S signal mediating their translocation to host cells^4,28^, a C-terminal activation domain activating host gene transcription^29^, two nuclear localization signals (NLS) directing them into host nucleus^30^, and a central repeat domain recognizing the target sequence by one repeat recognizing one nucleotide^31,32^. The TALE repeats have a similar composition and construction. A typical repeat is 34 aa long, forming a right-handed superhelix that can wrap around a double-stranded DNA^33,34^. The specification of a repeat is conferred by a pair of neighbored amino acids at position 12 and 13, called the repeat-variable diresidue (RVD), which is situated at the loop linking two helices. The 12th residue stabilizes the loop and projects it into the major groove, while the 13th residue contacts the base deciding the specificity of target recognition^35^. The specificities and efficiencies of RVDs are variable. Each RVD contacts one or more DNA bases and efficient activation requires a well-balanced composition of “strong” and “weak” RVD^36^. The common correspondence between RVDs and their preferred nucleotides are interpreted as NI:A, HD:C, NG:T, NH:G, NN: G/A, NS:A/C/G/T^30^. TALENs could be designed to target almost any given DNA sequence in theory, but there are two limitations in the design of TALENs. One is the requirement for a thymine at the 5′ end of the target sequence, which will be recognized by the two amino-terminal cryptic repeat folds^37^. The other is the fussy and labored procedure to construct a powerful editing plasmid. In spite of these, many companies have chosen this method to edit target gene for its high precision^38^.

In view of the facts that the *OsTFIIAγ5/Xa5* gene exists in the overwhelming majority rice varieties, the change of key amino acids in OsTFIIAγ5/Xa5 can break up its interaction with TALEs and the mutant *OsTFIIAγ5/Xa5* can improve the disease resistance mediated by *Xa21* or *Xa4* gene, we selected TALEN to edit the *TFIIAγ5* gene in popular breeding varieties and hope to improve their resistance to bacterial blight. Here we obtained many *TFIIAγ5* mutations of long-fragment deletion, substitution and insertion, and found that the TFIIAγ5 plays a double role in pathogen invasion, and the amino acids between the 32nd and 40th are critical for its function.

## Results

### TFIIAγ5 plays a role in the host infection of *Xoo*

**Figure 1:**
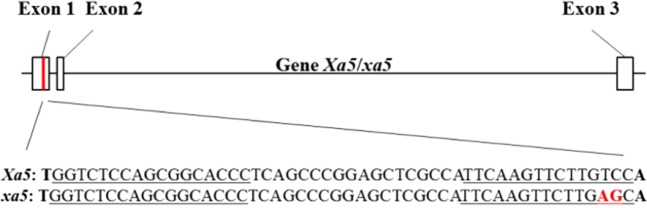
Structure of the gene *TFIIAγ5* (*Xa5*and*xa5*) displaying the discrepancy between the allelic *Xa5* and *xa5* that was involved in recognition by the TALEN-Xa5. The rice gene *TFIIAγ5* has two natural alleles, *Xa5* and *xa5*. The gene structure of both genes is the same and is shown above. The target region of the TALEN-Xa5 locates in the exon 1 and marked by a red strip. The underlined target sequence of TALEN-Xa5 in *Xa5* and *xa5* is showed in the bottom of the figure. The discrepancy of the two nucleotides between *Xa5* and *xa5* was also showed in red letters.

To assess whether the host TFIIAγ5 is required for the invasion of *Xoo*, we edited the *TFIIAγ5* using the TALEN method. The target sequence of our TALEN was designed from the first exon of *TFIIAγ5* to enhance the incidence of knock-out mutants (Fig. 1). The space sequence between the couple of target sequences is more mutable than the flanking regions, and enzyme restriction sites are normally designed here to detect the presence of mutations. Here, two restriction sites for *Bbv*CI and *Sac*I in the space sequence were employed to detect mutations by PCR restriction enzyme digestion assay (PCR-RE).

Considering the two nucleotides difference between *xa5* and *Xa5* (Fig. 1), the target sequence of the TALEN-Xa5 was designed to cover these two sites. The transient expression of the TALEN plasmid was first conducted in rice protoplasts containing *Xa5* (TP309) and *xa5* (IRBB5), respectively. Then the genome DNA was isolated from these protoplasts and subjected to the PCR-RE analysis. Sequence analyses of these PCR productions showed that the fragments from the *xa5* gene all kept the initial characteristics of *Bbv*CI and *Sac*I digestion (Supp. Fig. S1). The result showed that the TALEN-Xa5 plasmid specializes in the *Xa5* edition.

**Figure 2:**
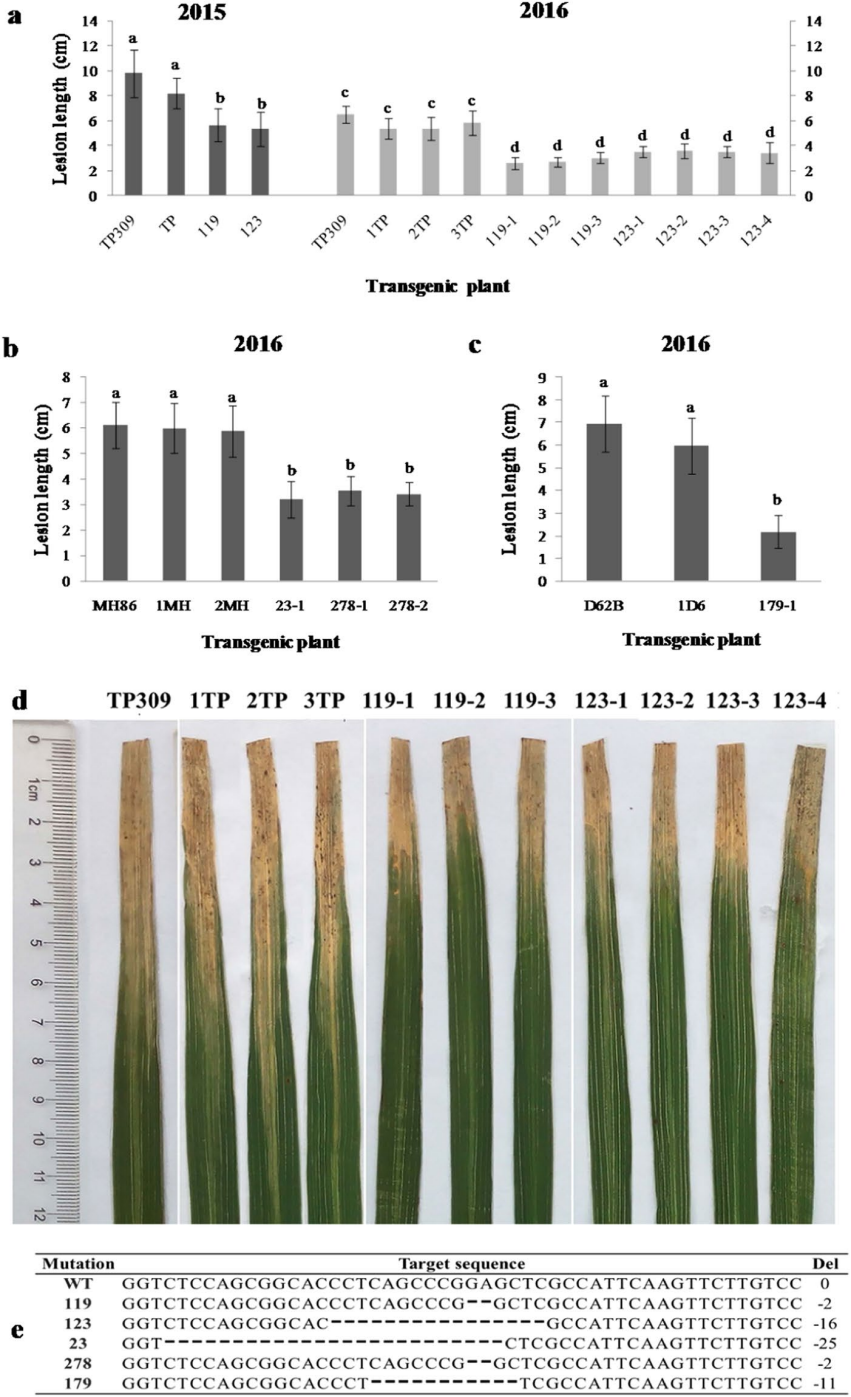
The lesions length of rice leaves in the knock-out mutants 14 days after inoculation with PXO86. (**a**) The T_2_ plants and their derivative T_3_ plants were inoculated by PXO86 in Beijing in August, 2015 and 2016 respectively. (**b**,**c**) The T_3_ plants in MH86 or D62B background were examined in Hainan in February, 2016. (**d**) The disease symptoms of the T_3_ plants were photographed 14 days after inoculation. (**e**) The targeted sequences of TALEN-Xa5 in transgenic plants. WT is the initial target sequence of *Xa5*, and the others are the mutant sequences. TP309, MH86, and D62B. TP309, MH86, and D62B are blank controls; TP, 1TP, 2TP and 3T were transgenic controls in TP309 background with pCAMBIA1300 vector; 1MH and 2MH are transgenic controls in MH86 background; 1D6 is the transgenic control in D62B background. All controls had the same target sequence as the WT. The other rice lines were knock-out mutants with their target sequences shown in e. TP, 119, 123, 278, 23 and 179 are the homozygous T_2_ mutant plants. 1TP, 2TP and 3TP are the random selected T_3_ plants derived from the TP; 119-1, 119-2 and 119-3 are the T_3_ plants from the 119; 123-1, 123-2, 123-3, and 123-4 are the T_3_ plants from the 123. The 278-1 and 278-2 are homozygous mutant T_3_ plants from the 278. 23-1 and 179-1 are homozygous mutant T_3_ plants from 23 and 179 respectively. Bars represent the average ± SD of three biological repeats. Different letters above columns indicate significant differences at *P* < 0.05 as determined by a one-way ANOVA followed by post hoc Tukey honest significant difference (HSD) analysis.

Subsequently, the TALEN-Xa5 plasmid was transformed into a rice callus by *Agrobacterium*-mediated genetic transformation and yielded many transgenic lines. We analyzed these transgenic seedlings through PCR and a sequencing method, and finally obtained T_1_ seedlings with homozygous frame-shift mutations of *Xa5*. Firstly, the *Fok*I gene was detected in these transgenic plants (see the PCR primer showed in Supp. Table S1). The absence of *Fok*I in T_1_ generation plants meant that their *Xa5* gene was not edited later. Secondly, the *Xa5* mutants in these plants without FokI were selected by PCR/RE analyses (see PCR primer showed in Supp. Table S1) and sequencing. Finally, the T_2_ transgenic plants with the homozygous frame-shift mutant *Xa5* were obtained and inoculated with the *Xoo* strain PXO86. Two of these (also referred to as the knock-out mutants), line 119 and line 123 deriving from the *Japonica* variety TP309, were checked for two consecutive generations in Beijing in 2015 and in Hainan in 2016 (Fig. 2a). The blank control was TP309 and the transgenic control was a line of the positive transgenic TP309 (TP), which did not contain the *Fok*I gene in the T_1_ generation and thus had an intact *Xa5* gene in the T_2_ generation. The lesion length of TP309 was 9.82 ± 1.90 cm in the experiment in Beijing and 6.51 ± 0.67 cm in the Hainan experiment. The lesion length of the TP plant was 8.20 ± 1.20 cm in the Beijing experiment, and the lesion length of the three T_3_ lines from the T_2_ transgenic control was 5.38 ± 0.82, 5.36 ± 0.93, 5.84 ± 0.97 cm, respectively, in the Hainan experiment. Apparently, although each line had a longer lesion length in the 2015 experiment, all mutation lines had a shorter lesion length than the controls. Similar results were obtained from the knock-out mutants of *Indica* varieties, MH86 (Fig. 2b) and D62B (Fig. 2c). All these results showed that the knock-out of *TFIIAγ5* alleviates the bacterial leaf blight disease in rice (Fig. 2), and TFIIAγ5 might facilitate *Xoo* in the host infection

### TFIIAγ5 can be used by different *Xoo* strains to infect rice

**Figure 3:**
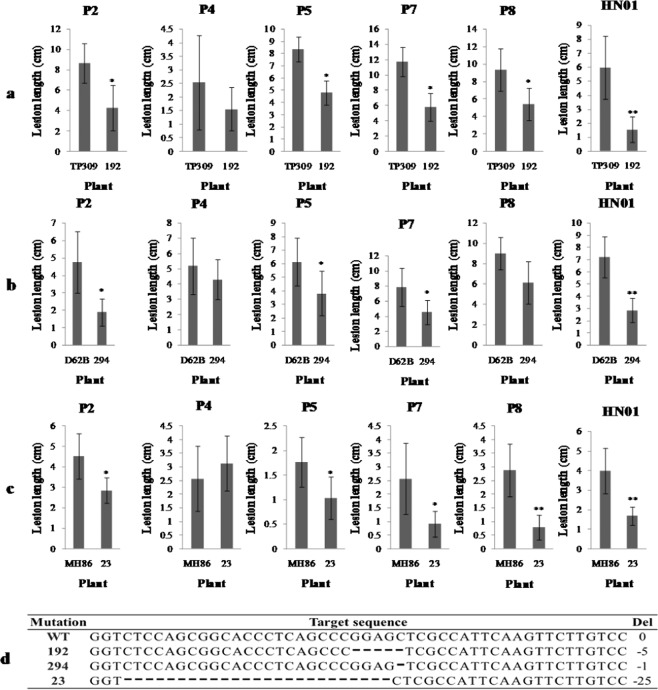
Resistance spectrum analyses of the *Xa5* knockout mutants. The spectrum analysis was carried out in TP309 (**a**), D62B (**b**), and MH86 (**c**) genetic backgrounds. (**d**) The targeted sequences of TALEN-Xa5 in transgenic plants. The target sequence in TP309, D62B, and MH86 are same and refer as WT; 192, 294 and 23 are the homozygous T_2_ knockout mutants from TP309, D62B and MH86 respectively. PXO86 (P2), PXO71 (P4), PXO112 (P5), PXO145 (P7) and PXO280 (P8) are the Philippines *Xoo* strains; HN01 is a newly isolated *Xoo* strain from Hainan, China. Bars represent the average ± SD of three biological repeats. Asterisks signs indicate a statistically significant difference compared with the control plants (*P < 0.05 and **P < 0.01).

When infecting a plant, bacteria induce and recruit host genes by secreting effectors into the host cells through the type III system. These effectors can interact with the host components to exert their function. To explore the function of TFIIAγ5 in the infection process, we analyzed the disease symptoms of these knock-out mutants by inoculating them with different *Xoo* strains (Fig. 3).

The TP309 mutant line 192 had a 5-nucleotide deletion in the first exon of *TFIIAγ5* (Fig. 3a), the D62B mutant line 294 had a 1-nucleotide deletion in the first exon (Fig. 3b), and the MH86 mutant line 23 had a 25-nucleotide deletion in the first exon (Fig. 3c).

Six *Xoo* strains were used to examine whether the TFIIAγ5 was recruited during infection. Five of the strains were isolated from the Philippines, namely PXO86 (P2), PXO71 (P4), PXO112 (P5), PXO145 (P7) and PXO280 (P8). The other strain was HN01, a new isolate from Hainan, which can overcome the resistance of *Xa21* in rice. The statistical analysis showed that the *Xa5* depletion decreased the rice’s susceptibility to *Xoo* with different genetic backgrounds (Fig. 3a–c). These indicated that the TFIIAγ5 can be used by different *Xoo* strains to infect rice.

### *Xa5* and *xa5* function differently in response to the invasion of *Xoo*

The severity of bacterial leaf blight depends on many factors, such as the growing environment and genetic background. However, the inoculation analyses in 2015 and 2016 year at two breeding bases in Beijing and Hainan exhibited a similar trend, namely that the knock-out mutant plants of *Xa5* showed enhanced resistance to *Xoo* than the control plants (Fig. 2). IRBB5 and IR24 are near-isogenic lines, and they respectively had *xa5* and *Xa5* in its *TFIIAγ5* loci (Fig. 1). It is a pity that we were not able to obtain the knock-out mutant from IR24, due to its poor capacity for regeneration after transformation. Thus, it is difficult to determine whether the high resistance of IRBB5 was brought about by *xa5* or merely by the genetic background.

**Figure 4:**
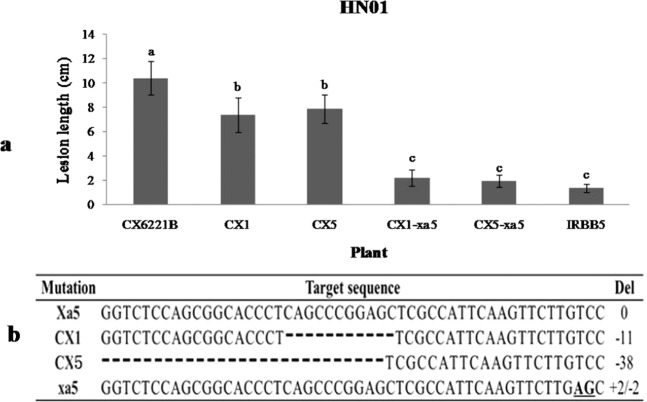
Resistance analysis of *Xa5* and *xa5* in the plants containing *Xa21* gene. (**a**)The lesions length of rice leaves in the knock-out mutants 14 days after inoculation with HN01. (**b**) The targeted sequences of TALEN-Xa5 in transgenic plants. CX1 and CX5 are T_3_ knock-out mutants from CX6221B (a stable hybrid line with *Xa21* gene in D62B background), CX1-xa5 and CX5-xa5 are the T_0_ transgenic plants with *xa5* gene in CX1 and CX5 background respectively. CX6221B contains *Xa5* and *Xa21* gene, while IRBB5 only has the gene *xa5*. The HN01 was able to overcome the resistance of *Xa21* gene, but not that of *xa5* gene. The standard deviation (STDEV) is indicated in each column. Bars represent the average ± SD of three biological repeats. Different letters above columns indicate significant differences at *P* < 0.05 as determined by a one-way ANOVA followed by post hoc Tukey honest significant difference (HSD) analysis.

To further clarify the function of TFIIAγ5 in pathogen infection, *xa5* was transformed into two knock-out mutants of *TFIIAγ5*, CX1 and CX5 by the agrobacterium-mediated method. CX1 and CX5 are from the rice breed CX6221B, which carries the resistant gene *Xa21* in the genetic background of the rice D62B^39^. The *TFIIAγ5* lacks 11 and 38 nucleotides in the first exon respectively in these two mutants (see the bottom of the Fig. 4). At least 20 T_0_ positive transgenic lines for each transformation were obtained. These transgenic plants carrying the *xa5* gene were named CX1-xa5 or CX5-xa5. In 2015, they were inoculated with the *Xoo* strain HN01, which was able to overcome the resistance of *Xa21* in rice. All these plants showed significantly enhanced disease resistance, and have shorter lesions of leaves than the two wild type plants and the *TFIIAγ5* knock-out mutants (Fig. 4). These indicated that the *Xa5* and *xa5* function differently in response to the invasion of pathogens.

### The amino acids around 32nd site are important for TFIIAγ5 in the response to *Xoo*

**Figure 5:**
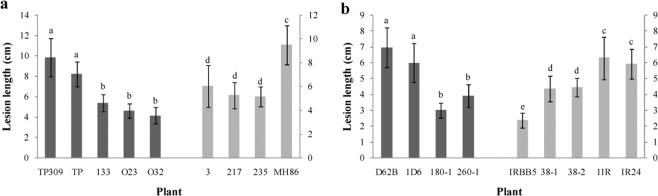
Lesion length in rice leaves of in-frame mutants inoculated with *Xoo*. (**a**) The homozygous T_2_ plants of in-frame mutants were inoculated by PXO86 in Beijing in August, 2015. TP309 and MH86 were the control plants containing the gene *Xa5*; TP is the T_2_ transgenic control plant from TP309 with pCAMBIA1300 vector. 133 is the T_2_ plant from TP309 with mutant Xa5 protein, in which two amino acids were replaced with six amino acids in the exon 1 (type 3 in the Table 1). The mutant Xa5 proteins in O23, O32 and 3 all have an amino acid deletion in the exon 1 (type 1 in Table 1). 217 expresses a mutant Xa5 protein with four amino acid-deletion in the exon 1 (type 5 in Table 1). 235 expresses the mutant Xa5 protein, in which five amino acids were replaced with nine amino acids in the exon 1 (type 6 in the Table 1). IRBB5 contains the homozygous resistant gene *xa5*, and functions as the resistant control here. (**b**) The homozygous T_3_ plants of in-frame mutants from D62B and IR24 were inoculated with PXO86 in Hainan in February 2016. D62B and IR24 are the rice varieties with homozygous *Xa5* gene. 1D6 and 1IR are the T_3_ transgenic control plants with pCAMBIA1300 plasmid derived from D62B and IR24 respectively. 180-1 expresses a mutant Xa5 protein with three amino acid-deletion in the exon 1 (type 4 in Table 1). 260-1 expresses a mutant Xa5 protein with an amino acid insertion in the exon 1 (type 8 in Table 1). 38-1 and 38-2 are the T_2_ plants derived from 38 that have a nine amino acid-deletion in the exon 1 of the Xa5 protein (type 2 in Table 1). Bars represent the average ± SD of three biological repeats. Different letters above columns indicate significant differences at *P* < 0.05 as determined by a one-way ANOVA followed by post hoc Tukey honest significant difference (HSD) analysis.

In the TALEN-Xa5 transformation, eight types of in-frame mutants were generated besides the knocked-out mutants of *Xa5* (Table 1). All the edited sites in these mutants took place in the region between the 26th and the 40th amino acids of TFIIAγ5. These mutations were then tested for their resistance to *Xoo* in the T_2_ or T_3_ homozygous plants lacking intact TALEN-Xa5 proteins. For example, the type 1 mutation lacked the 32nd amino acid and the type 8 was inserted by a glutamic acid (E) between the 32nd and the 33rd amino acid. The lesion length of the mutants of type 1 was significantly shorter than that of their wild type varieties (Fig. 5a). The lesion length of leaves in the mutant 3 from MH86 was 6.00 ± 1.09 cm; the length of the lesions on leaves in the mutants O23 and O32 from TP309 were 4.61 ± 0.72 and 4.14 ± 0.79 cm, respectively. However, the lesion length of leaves in MH86 was 9.47 ± 1.78 cm, and that of TP309 was 9.82 ± 1.90 cm. Moreover, the lesion length of leaves in these type 1 mutant plants were similar to that in the other types of in-frame mutant plants in each background. The length of the lesions on leaves in the type 7 mutant 217 and type 8 mutant 235 from MH86 were 5.24 ± 0.84 and 5.15 ± 1.65 cm, respectively, similar to that of mutant 3. The lesion length of the leaves in the type 3 mutants 133 was 5.36 ± 0.86 cm, similar with that of the mutants of O23 and O32 (Fig. 5a). The knockout lines 119 and 123 were 5.65 ± 1.32 and 5.32 ± 1.35 cm, respectively, similar to the in-frame lines with the TP309 background (Supp. Fig. S2a). The type 8 mutant 260-1 from D62B harbored an amino acid insertion between the 32nd and 33rd amino acids. The lesion length of its leaves was 3.9 ± 0.73 cm, which was shorter than that of D62B, which was 6.95 ± 1.25 cm (Fig. 5b). However, it was slightly longer than that in the type 4 mutant 180-1 (2.97 ± 0.49 cm) and the knocked-out mutant 179-1 (2.17 ± 0.73 cm) with the same background (Supp. Fig. S2b). The type 4 mutant 180-1 lacked the three amino acids from the 32nd to the 34th amino acid of the Xa5 (Table 1). All these results indicated that the amino acids around the 32nd site might be crucial to TFIIAγ5 in governing rice’s susceptibility to *Xoo*.

**Table 1:** The DNA and protein sequencesof the gene *TFIIAγ5* in in-frame mutants edited by TALEN-Xa5.

No.	Target sequence of TALEN-Xa5	Indels
*Xa5*	GATGGTCTCCAGCGGCACCCTCAGCCCGGAGCTCGCCATTCAAGTTCTTGTCCAGTTT	—
1	GATGGTCTCCAGCGGCACCCTCAGCCCG---CTCGCCATTCAAGTTCTTGTCCAGTTT	−3
2	GATGGTCTCC---------------------------ATTCAAGTTCTTGTCCAGTTT	−27
3	GATGGTCTCCAGCGGCACCCTCAGCCCGG---T----A---GAG--CTTGTCCAGTTT	−17/+5
4	GATGGTCTCCAGCGGCACCCTCAGCCC---------CATTCAAGTTCTTGTCCAGTTT	−9
5	GATGGTCTCCAGCGGCACC------------CTCGCCATTCAAGTTCTTGTCCAGTTT	−12
6	GATGGTCTCCAGCGGCACCCTCAGCCCG-CGTGCGCC--T-AA--AC-T-T----TTT	−27/+15
7	--------------------------------CTCGCCATTCAAGTTCTTGTCCAGTTT	−39
8	GATGGTCTCCAGCGGCACCCTCAGCCCGGAG/CTCGCCATTCAAGTTCTTGTCCAGTTT	+3GAG
*xa5*	GATGGTCTCCAGCGGCACCCTCAGCCCGGAGCTCGCCATTCAAGTTCTTGAGCAGTTT	−2/+2
**No**.	**First 50 amino acids of TALEN-Xa5**	**changes**
Xa5	MATFELYRRSTIGMCLTETLDEMVSSGTLSP**E**LAIQVLVQFDKSMTEALE	—
1	MATFELYRRSTIGMCLTETLDEMVSSGTLSP-LAIQVLVQFDKSMTEALE	32D
2	MATFELYRRSTIGMCLTETLDEMVS---------IQVLVQFDKSMTEALE	26–34D
3	MATFELYRRSTIGMCLTETLDEMVSSGTLSP---**VE**---LVQFDKSMTEALE	32–37D
4	MATFELYRRSTIGMCLTETLDEMVSSGTLSP---IQVLVQFDKSMTEALE	32–34D
5	MATFELYRRSTIGMCLTETLDEMVSSGT----LAIQVLVQFDKSMTEALE	29–32D
6	MATFELYRRSTIGMCLTETLDEMVSSGTLSP--**RAPKL**--FDKSMTEALE	32–40R
7	MATFELYRRSTIGMCLTET-------------LAIQVLVQFDKSMTEALE	20–32D
8	MATFELYRRSTIGMCLTETLDEMVSSGTLSPE/LAIQVLVQFDKSMTEALE	32–33I
xa5	MATFELYRRSTIGMCLTETLDEMVSSGTLSPELAIQVL**E**QFDKSMTEALE	39R

In a previous study, the 39th amino acid was characterized as a critical site of Xa5^13^. The 39th amino acid substitution of Xa5 leads it to be a recessive resistance protein xa5. Here we obtained two in-frame mutants 38-1 and 38-2 from IR24, they both had the deletion from the 26th to the 34th amino acids (type 2 in Table 1). IR24 containing the *Xa5* gene was the near isogenic line of resistant variety IRBB5 that harbors the homozygous *xa5* gene. The two mutant plants were inoculated with the *Xoo* strain PXO86 and investigated after two weeks. The lesion length of leaves in IR24 was 5.91 ± 0.93 cm and in IRBB5 this was 2.36 ± 0.47 cm. The length of the lesions on leaves in the mutants 38-1 and 38-2 were 4.36 ± 0.82 and 4.44 ± 0.58 cm, respectively, and were shorter than that of IR24 but longer than that of IRBB5 (Fig. 5b), indicating that the mutant Xa5 of type 2 enhance rice resistance, though the resistance is not as strong as that conferred by xa5. All these suggested that the amino acids around 32nd site are the critical amino acids determining TFIIAγ5’s action.

### TALEN-Xa5 can mediate off-target modification to the *TFIIAγ1* gene

**Figure 6:**
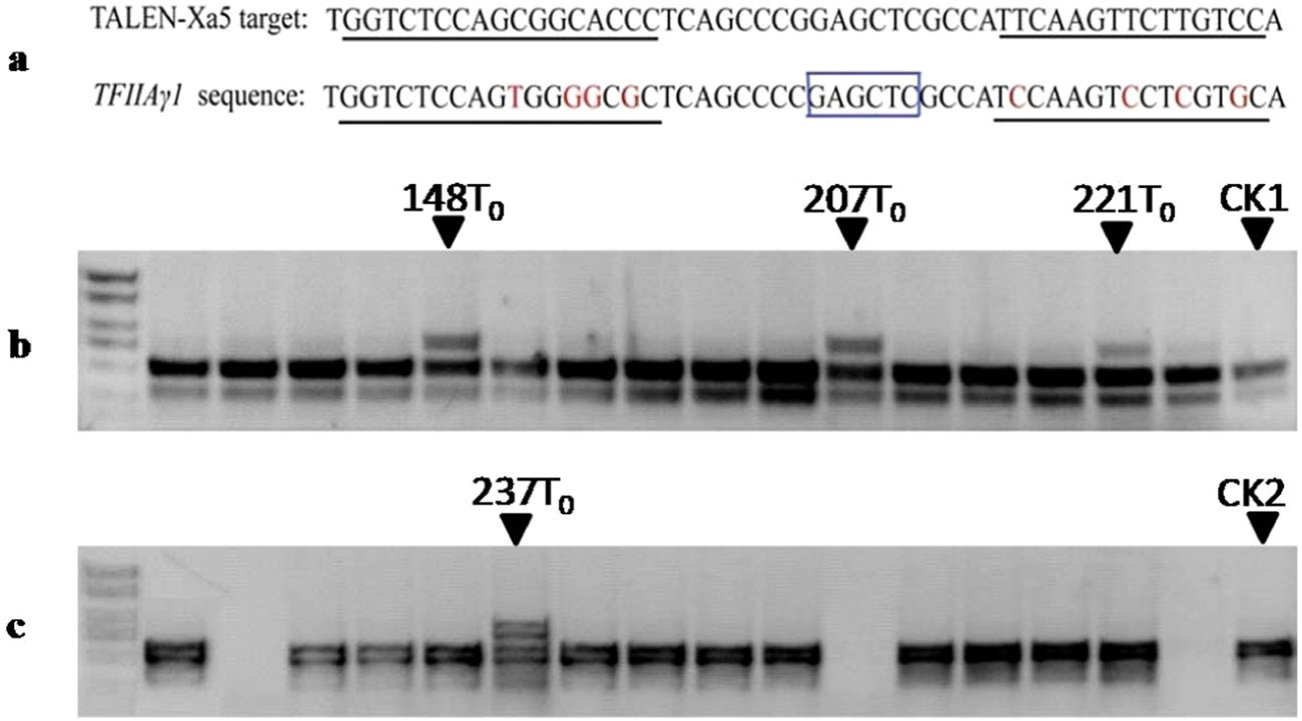
The off-target analysis of TALEN-Xa5 in the rice *TFIIAγ1* gene. (**a**) The possible target region of TALEN-Xa5 in the rice *TFIIAγ1* gene. The target sequence in the gene *Xa5* and the possible target sequence in the gene *TFIIAγ1* were underlined. The restriction enzyme cutting site of *Sac*I in the space region used to detect the mutations was marked with a blue box. The *Sac*I can cut the PCR product into two fragments of 511 bp and 302 bp in theory. (**b**) The T_0_ generation plants of TALEN-Xa5-transformed lines in TP309 background were examined by PCR/RE. 148T_0_, 207T_0_ and 221T_0_ are the plants with the mutant *TFIIAγ1* gene. CK1 is TP309 plant. (**c**) The T_0_ generation plants of TALEN-Xa5-transformed lines in MH86 background were examined by PCR/RE. 237 T_0_ is the plant with the mutant *TFIIAγ1* gene. CK2 is MH86 plant. The primers of TFIIA-gamma-1F and TFIIA-gamma-1Rwere used to examine off-target activity in *TFIIAγ1* were shown in Supp. Table S2.

There are two *TFIIAγ*-like genes, *TFIIAγ1* and *TFIIAγ5* in the rice genome. Sequence alignment showed that the sequence identity between *TFIIAγ1* and *TFIIAγ5* was 85.8% at the protein level and 81.3% on the cDNA level (Supp. Fig. S3). The possible off-target sequence in *TFIIAγ1* was determined through analyzing the sequences of *TFIIAγ1* and *TFIIAγ5*. Four mismatched sites were found in each of the two target regions (Fig. 6). Because the experiment of transiently expressing TALEN-Xa5 in rice protoplasts showed that the two nucleotides of difference in the target sequence led to undetectable edition in the gene *Xa5* (Supp. Fig. S1), the off-target detection of TALEN-Xa5 on the *TFIIAγ1* was only conducted in the *Xa5* mutant lines for the definite editing activity. The cutting site (GAGCTC) for the restriction enzyme *Sac*I in the two target sequences was used to identify the off-target in *TFIIAγ1* through PCR/RE analysis (Fig. 6). The PCR product for the potential target sequence of *TFIIAγ1* can be digested by *Sac*I into a band of 302 bp and a band of 511 bp. In the 49 T_0_ lines containing edited *TFIIAγ5*, only four lines were detected to have edited *TFIIAγ1* by the PCR/RE analysis, indicating that the high degree of nucleotide sequence similarity between TFIIAγ5 and TFIIAγ1 can lead to the off-target of TALEN-Xa5 on the *TFIIAγ1* though the off-target efficiency is very low (Fig. 6). In addition, the four *TFIIAγ1* mutants were all chimeras or heterozygotes and had many various mutations besides the wild type of *TFIIAγ1* (Supp. Table S2). It is regrettable that none of the four *TFIIAγ1* mutants survived for the T_1_ generation; one died in the seedling stage and the others were sterile.

### The disease resistance mediated by *Xa21* was independent of *TFIIAγ5*

**Figure 7:**
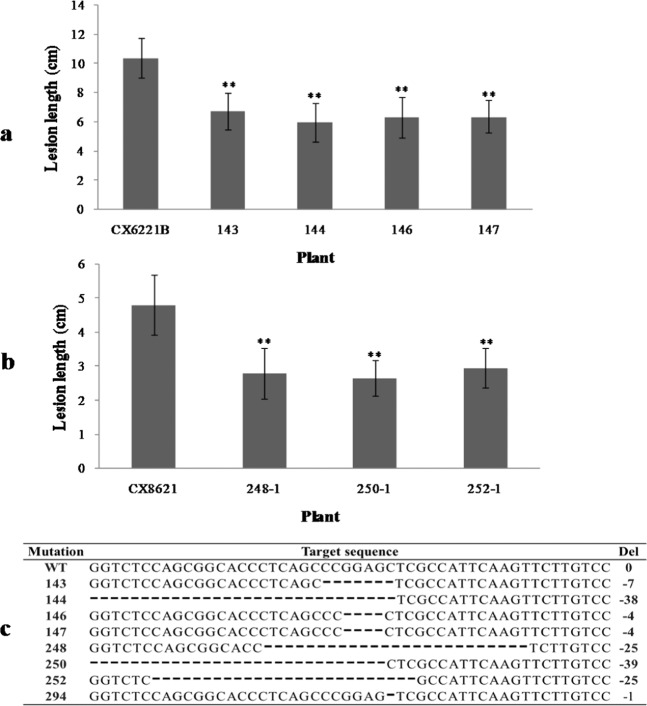
Lesion lengths in the leaves of *TFIIAγ5/Xa5* mutant plants with *Xa21* gene. (**a**) Inoculation analysis of the T_2_ mutant plants from CX6221B was conducted in Beijing in August, 2015. (**b**) Inoculation analysis of the T_3_ mutant plants from CX8621 was carried out in Hainan in February, 2016. 248-1, 250-1 and 252-1 are the T_3_ homozygous mutants in CX8621 background from the T_2_ plants 248, 250 and 252, respectively. (**c**) The targeted sequences of TALEN-Xa5 in transgenic plants. CX6221B and CX8621 are the plants contain the homozygous resistant gene *Xa21* derived from the varieties D62B and MH86 respectively. 143, 144, 146, and 147 are the T_2_ mutants in CX6221B background. D62B, CX6221B and CX8621 have the wild type *Xa5*, shown as WT in the bottom table. All the plants were inoculated with *Xoo* strain, HN01. Bars represent the average ± SD of three biological repeats. Asterisks signs indicate a statistically significant difference compared with the control plants (*P < 0.05 and **P < 0.01).

*Xa21*, encoding a receptor kinase-like protein, confers on rice a broad-spectrum resistance to multiple pathogen isolates of *Xanthomonas oryzae* pv. oryzae. It is a dominant gene and is usually used together with other resistant genes such as *xa5* in breeding for permanent and stable disease resistance. In this study, we also edited *Xa5* in the plants with the *Xa21* gene, CX6221 and CX8621^39^. They were derived from the two widely used restorer lines D62B and MH86 by integrating a single copy of *Xa21*. Four homozygous mutant Xa5 T_2_ lines and three homozygous Xa5 mutant T_3_ lines were obtained, respectively, in CX6221 and CX8621. Most of these mutant lines were knocked-out mutants except for the line 250-1 from CX8621 (the type 9 mutation in Table 1) that lacked 13 amino acids from the 19th to the 32nd of Xa5. To analyze the resistance phenotypes, these mutants were cultured and inoculated with the *Xoo* strain HN01 at the tillering stage in Beijing or Hainan (Fig. 7a,b). The HN01 was isolated from Hainan and can overcome the disease resistance conferred by *Xa21*, but not *xa5*.

The length of the lesions on the leaves in the four mutants from CX6221B ranged from 5.98 ± 1.32 to 6.75 ± 1.24 cm, indicating that they have a uniform phenotype (P > 0.05) and are significantly shorter (P < 0.01) than that of the control plant CX6221B (about 10.41 ± 1.37 cm). The same results were achieved from the mutant CX8621 plants. The length of the lesions on the leaves in 248-1, 250-1 and 252-1 were 2.80 ± 0.75, 2.65 ± 0.52, 2.95 ± 0.59 cm, respectively, and are shorter than that of CX8621(4.81 ± 0.87 cm). These results were similar to the experiment on the knocked-out mutants of *Xa5* inoculated by PXO86 (Fig. 2). In the same way, these mutant plants were inoculated with other Xa21-incompatible *Xoo* strains, PXO99 (P6), PXO71 (P4), PXO112 (P5), PXO145 (P7), and PXO280 (P8). These mutant plants showed a similar phenotype to the control plants, and the lengths of the lesions on their leaves were all less than 1 cm. This indicated that the knocked-out mutants of Xa5 all displayed a similar reduced effect on the disease symptoms of rice plants with or without Xa21, though climate and environment may have some influence on disease development (Fig. 7a,b).

## Discussion

Bacterial blight caused by *Xoo* is one of the most destructive rice diseases throughout the world, and stands out in the top 10 bacterial diseases (Mansfield *et al*., 2012). In some areas of Asia, this disease can reduce crop yield by up to 50%. The most effective approach to combat it is the use of resistant varieties (Khush *et al*. 1989). More than 40 bacterial blight resistance genes have been identified in rice and ten of them have been cloned and characterized (Kim *et al*. 2015; Hutin *et al*. 2015). DNA sequence and function studies have showed that the encoding proteins of these cloned genes are structurally diverse, indicating that their resistance mechanisms to bacterial blight are very complicated (Song *et al*. 1995). To date, only a few of these genes have been used for resistance breeding (Hutin *et al*. 2015). Generally, the dominant resistance genes have been paid the most attention in breeding. For example, numerous resistant breeding lines harboring the dominant resistance gene *Xa4* have been developed since the first resistant varieties, IR20 and IR22, were released in 1969 (Khush GS, 1977). In China, the *Xa4* gene has also been widely integrated into the parental lines of hybrid rice since 1980 (Zhang, 2009). The first map-based cloned broad-spectrum resistance gene, *Xa21*, was introduced into many rice varieties and other plants through agrobacterium-mediated or marker-assisted breeding method (Datta *et al*. 2002; Huang *et al*. 1997; Kottapalli *et al*. 2010; Luo and Yin 2013; Luo *et al*. 2014; Perez *et al*. 2008; Rajpourohit *et al*. 2011; Singh *et al*. 2001; Zhang *et al*. 2006; Zhai *et al*. 2000; Tripathi *et al*. 2014). However, these dominant genes are vulnerable to counter the evolution of pathogens (Lee *et al*. 1999). Comparatively speaking, the resistance conferred by recessive genes is much more stable, but the recessive properties limit their application in rice breeding through conventional methods or plant biotechnology approaches. In this study, we used TALEN-based techniques to edit the dominant allelic of the *xa5* gene and obtained a serial of mutant *Xa5*/ *TFIIAγ5* genes with different rice backgrounds. In the T_0_ or T_1_ transgenic plants, most were able to reduce rice susceptibility to varying degrees, indicating that the TALEN-based editing technique is very efficient in accelerating the application of the *xa5* gene in rice breeding.

The *xa5* gene was first characterized in the rice varieties IR1545-284 and RP291-7 (Petpisit *et al*. 1977). It confers rice a broad-spectrum resistance to *Xoo* strains, including the Philippine races 1–3 and all Japanese races. Previous studies showed that the pyramid lines of the xa5 gene and some other dominant genes such as Xa21 and Xa4 have a higher and wider spectrum resistance than the plants harboring only one resistance gene (Huang *et al*. 1997). However, there is an exceptional case, where the resistance mediated by *Xa27* depends on the *Xa5* (*OsTFIIAγ5*) instead of *xa5* (*OsTFIIAγ5*^*V39E*^)^5,22,24^. The current model of Xa5 is that it interacted directly with the TAL effector of *Xoo* or *Xoc* to cause bacterial blight or streak^21^. The *xa5* is correlated with the reduced expressions of OsSWEET genes, which led to a virulence effector-dependent quantitative trait for bacterial blight^24^. To probe the particular function of Xa5, we edited it through the TALEN-based technique and obtained many mutants including in-frame and knock-out mutants. These edited *Xa5* mutants enhanced rice resistance to *Xoo* (Fig. 2 and Supplementary Fig. S2), but their resistance was inferior to that conferred by the resistant gene *xa5* (Figs. 2 and 4). When these mutant plants were transformed with the *xa5* gene, they were similar to IRBB5 in disease resistance. These results indicated that the mutant Xa5 with 39th amino acid replacement has a different effect from that with the amino acid deletion or insertion around 32nd site in response to *Xoo*. However, the previously study showed that the amino acids between 31st and 50th form the domain of alpha-helices 3 (H3) in Xa5^13^, the amino acids in the domain are logical to have the same effects. We speculated that the different effects are due to the amino acids deletion or insertion of Xa5 can destroy H3 domain, while the amino acid replacement just change the conformation or binding property of Xa5.

Previous studies showed that TALEN-mediated genome modification is accompanied by very rare off-target effects^40,41^. In this study, among the 49 T_0_ transgenic lines containing edited *TFIIAγ5*, four lines were detected to have edited *TFIIAγ1* by the PCR/RE analysis, showing that TALEN can mediate off-target modification though the efficiency is very low. *TFIIAγ1* and *TFIIAγ5* were speculated to arise from whole genome duplication in the common ancestor of grasses, and share a high degree of nucleotide sequence similarity with each other^13,42^. The high degree of nucleotide sequence similarity between TFIIAγ5 and TFIIAγ1 is the main reason for causing the off-target of TALEN-Xa5 on the *TFIIAγ1*. In addition, unlike the RNAi and Cas9-edited *TFIIAγ1* transgenic plants^21,43^, none of the four TALEN-edited *TFIIAγ1* plants survived for the T_1_ generation; one died in the seedling stage and the others were sterile. The lower off-target efficiency and the random insertion of T-DNA may account for these. The lower off-target efficiency leads to the lower yield of TALEN-edited *TFIIAγ1* plants, and the insertion of T-DNA can destroy some important genes to result in plant sterile. So the smaller sample capacity can not reflect the real effect of TALEN-edited *TFIIAγ1*. We will probe the TALEN-edited *TFIIAγ1* based on its own sequence in the future.

## Methods

### Construction of the TALEN-Xa5 expression vectors

The target selection of TALEN-Xa5 considered some qualifications described by a series of researchers and employed a web-based tool called TAL Effector-Nucleotide Targeter 2.0 (TALE-NT 2.0; https://boglab.plp.iastate.edu/)^44^. In addition, we restricted the pair of target sequences to the first exon of the *Xa5* gene.

The central repeat domain of TALEN was constructed and then inserted into a commercial TALEN skeleton (Sidansai, Shanghai, China) of clone plasmid using enzyme digestion and a ligation reaction. The two TALEN-Xa5 monomers were respectively generated using the different promoters 35S and Ubiquitin, while they employed the same nopaline synthase (NOS) terminator. Two cassettes of monomer genes were tandemly linked up by an *Asc*I site, and then were inserted into the expression vector of pCAMBIA1300 (GenBank accession number AF234296.1) after the *Kpn*I/*Sac*I digestion. Generally, the construction of the TALEN-Xa5 vectors followed the instructions of the Sidansai commercial kit. Finally, the accuracy of the TALEN-Xa5 vector was examined by Sanger sequencing at each construction step.

### *Agrobacterium*-mediated transformation of rice

The binary plasmid containing the TALEN-Xa5 in the pCAMBIA1300 was introduced into *Agrobacterium* by electroporation and then the *Agrobacterium* infection of calluses transmitted the expression vectors into rice.

Calluses of *Japonica* and *Indica* cultivar were generated from seeds and transformed by *Agrobacterium tumefaciens* LBA4404 and EHA105, respectively^45^. Four recipient rice cultivars were used in the experiment. TP309 belongs to *O. sativa Japonica* while MH86, D62B and IR24 belong to *O. sativa Indica*. Moreover, two transgenic lines of the gene *Xa21* created by our lab were used for a combination test of the resistance loci of the gene *Xa21* and *xa5*. The transgenic lines of CX8621 and CX6221 were the F4 or F5 generations of *O. sativa* cultivar MH86 and D62B, respectively. The primers Hpt-F and Hpt-R for the *HPT* gene were used to select the positive seedlings in the T_0_ generation (Table S1).

### The detection of mutations by PCR/RE assay and sequencing in transgenic rice

The transgenic seedlings were maintained in sterile conditions and checked for mutations before transplantation to the soil. The mutation detection was carried out using the PCR/RE assay and was further proved using gene sequencing as described^27^. The genomic DNA of transgenic seedlings was extracted following the CTAB-DNA precipitation method, providing the template for amplifying the fragment containing the TALEN-Xa5 target site in the PCR/RE assay. The PCR primers were showed in Table S1 as Xa5F and Xa5R. The amplification product was then digested by two restriction endonucleases *Bbv*CI and *Sac*I. The sites of *Bbv*CI and *Sac*I were respectively located at each end of the space lying between the TALEN-Xa5 target pair. If the TALEN-Xa5 edited the two endonuclease sites, distinct gel electrophoresis strips would appear, indicating the occurrence of mutations. In some cases, however, mutations occurred beyond the space region, leading to the loss of mutants in the PCR/RE assay. Nevertheless, missing a mutant is rare, and the mutation rate dropped down sharply away from the TALEN cut site in the space region. The mutations proved by PCR/RE were then verified by sequencing.

Several types of mutations frequently existed in one seedling, especially for the first transgenic generation. In this case, each mutation band from the PCR/RE digestion was purified and cloned to into the pEASY-T1 vector, and then heat shock transformed competent cells of *E. Coli* before sequencing plasmids were conducted and five or six positive clones of each transformation were randomly selected for the Sanger sequencing. The occurrence of mutations indicated gene editing on the gene *Xa5* in the transgenic seedlings. Only *Xa5* mutants in the T_0_ seedlings were reserved to produce T_1_ generation.

### Screening the separation of transgenic fragments in T_1_ generation

The genomic DNA from individual rice seedlings was extracted using the CTAB-DNA precipitation method. *Fok*I genes in each monomer of the *TALEN-Xa5* and the *HPT* gene in the expressing vector skeleton were screened using PCR to examine whether they had segregated. The detection primers for each gene were shown as Fok-F and Fok-R, Hpt-F and Hpt-R in Table S1. Notably, the mutations of gene *Xa5* were checked by the PCR/RE assay in the seedlings of the T_1_ generation that tested negative for the *Fok*I and *HPT* genes. Only *Xa5* mutants negative for the *Fok*I and *HPT* genes were maintained to produce T_2_ generation.

### Screening homozygous mutants of the gene *Xa5* in T_2_ generation

The PCR/RE assay and Sanger sequencing were used to recheck the *Xa5* mutants within T_2_ generation. Five or more seedlings of every T_2_ line were randomly selected. Homozygous mutants identified by the PCR/RE assay were then to be sequenced. The PCR product from the homozygous mutant was firstly purified and cloned to into the pEASY-T1 vector, and then transformed into competent cells of *E. Coli*. Five or six positive clones from each transformation were selected randomly for the Sanger sequencing. All positive clones showing the same type of mutations indicated that a seedling was a homozygous mutant. Only T_2_ homozygotes for the gene *Xa5* mutation were used to generate T_3_ homozygous lines. Additionally, some T_2_ lines from the homozygotes of the T_1_ mutants were already homozygous. It was important that all samplings from the same line showed the same mutation.

### Inoculation test in homozygous lines of gene *Xa5* mutations

Only homozygous lines were used in the inoculation test of the bacterial blight pathogen to assess the effect of gene *Xa5* mutations on rice resistance, which may be exerted in the T_2_ or T_3_ generation. The inoculation test was carried out two times. The first time, rice was grown during the normal growing season, from April to October in 2015 in Beijing (39°N latitude), China, then inoculated in July, with data collection in August. The second time, rice was grown from November, 2015 to April, 2016 under field conditions in Lingshui (18°N latitude), Hainan, China, then inoculated in January with data collection in February.

For the resistance evaluation, the *Xoo* strains PXO86 and HN01 (isolated in Hainan) were cultivated on PSA medium (30% potato filtrate, 0.05% Ca(NO_3_)_2_ • 4H_2_O, 0.2% Na_2_HPO_4_ • 12H_2_O, 1.5% sucrose, and 1.5% agar) at 28 °C for 2 days and then re-suspended in sterile water with a dilution of OD600 nm = 0.5 for inoculation. The cryopreserved strains should be activated on the PSA medium at 28 °C for 2 days and subsequently transferred to a new PSA medium. At least 10 plants of each line were examined. For the resistance spectrum assay, the *Xoo* strains PXO86, PXO71, PXO112, PXO99, PXO145, PXO280 and HN01 were employed. At least five plants from each line were examined. Five or ten leaves of each plant were inoculated using the leaf-clipping method for the booting seedlings (during the panicle development). Disease severity was determined on the basis of lesion length on the 14th day after inoculation. Significant differences between wild type controls and transgenic plants as determined by a one-way ANOVA (p < 0.05) followed by post hoc Tukey HSD analysis. All of the experiments were repeated two times with similar results.

### Detecting off-target effects on the homologous gene *TFIIAγ1*

Off-target effects on *TFIIAγ1*, another *TFIIAγ* gene in rice, were examined by PCR/RE. TFIIAγ1 is a paralog of rice TFIIAγ5 with 86% identity at the amino acid sequence level. The homologous off-target loci of the gene *Xa5* were blasted in National Center for Biotechnology in formation (NCBI)(https://blast.ncbi.nlm.nih.gov/Blast.cgi). PCR was performed using the primer TFIIA-gamma-1F and TFIIA-gamma-1R (Table S1). The restriction endonuclease *Sac*I was used to digest the amplification product. The normal product was digested into two fragments, one of 511 bp and the other 302 bp. The off-target effect of TALEN-Xa5 on *TFIIAγ1* might cause abnormal digestion fragments for the disappearance of the digestion site within the loop between the two target recognition sequences, providing a convenient way to evaluate the accuracy of the editing of TALEN-Xa5.

## Supplementary information

Supplementary information
