# Supplementary material for: TALEN-based editing of TFIIAy5 changes rice response to Xanthomonas oryzae pv. Oryzae
Source: Sci Rep. 2020 Feb 6;10:2036. doi: 10.1038/s41598-020-59052-w (PMC7005142; doi:10.1038/s41598-020-59052-w)
Supplement: Supplementary file 1 — Supplementary information [file 41598_2020_59052_MOESM1_ESM.pdf]

# **TALEN-based editing of *TFIIAy5* changes rice response to *Xanthomonas oryzae* pv. *Oryzae***

Jin Han [1,*], Zhihui Xia [2,*], Pengcheng Liu [1], Chunrong Li [1], Yanyan Wang [1], Lequn Guo [1], Guanghuai Jiang [1] & Wenxue Zhai [1]

[1]Institute of Genetics and Developmental Biology, Chinese Academy of Sciences, Beijing 100101, China

[2]Institute of Tropical Agriculture and Forestry, Hainan University, Haikou 570228, China

*These authors contributed equally to this work.

Correspondence and requests for materials should be addressed to W.Z. (wxzhai@genetics.ac.cn) or G.J. (email: ghjiang@genetics.ac.cn)

## Supplementary Information

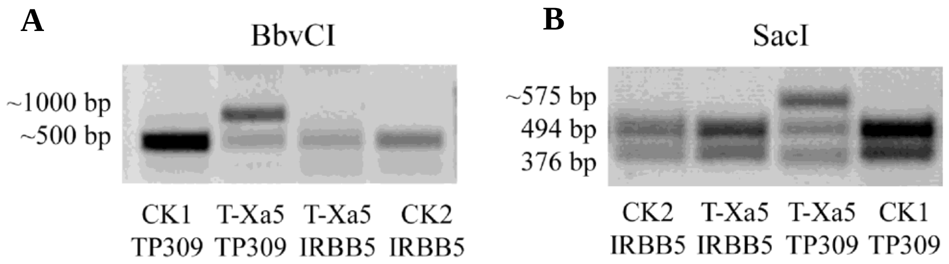


**Figure S1. Gene editing of TALEN-Xa5 on *Xa5* and *xa5* in rice protoplasts through transiently expression.** The detection was performed using PCR/RE. The primers of Xa5F and Xa5R (shown in Supp. Table S1) were used to amplify the target region, and two restriction endonucleases, *Bbv*CI (A) and *Sac*I (B), were selected to detect the sequence changes in the target region of TALEN-Xa5. T-Xa5 indicates the cell was transformed with TALEN-Xa5 plasmid, while the cell of CK1 and CK2 use a GFP reporter plasmid as a positive control of transformation and a negative control of gene editing respectively. Rice TP309 possesses the homozygous *Xa5* gene; however IRBB5 contains the homozygous *xa5* gene. The extra bands in the PCR product digested by *Bbv*CI (A) or *Sac*I (B) indicated the sequence changes in target region of TALEN-Xa5. Gene editing only takes place in TP309 protoplasts transformed with TALEN-Xa5.

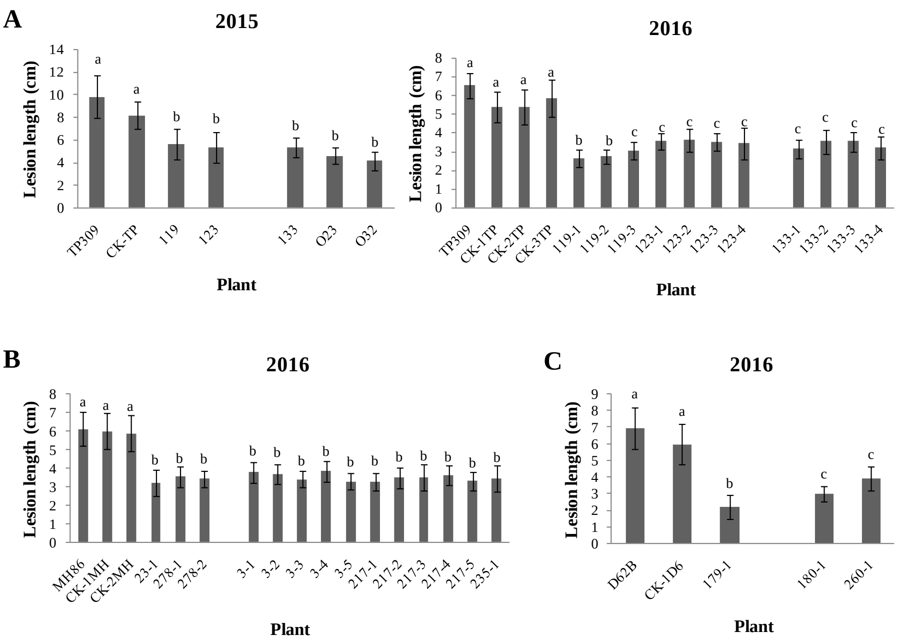


**Figure S2. The lesion length of the knockout mutants and in-frame mutants derived from TP309 (A), MH86 (B) and D62B (C).** The experiments were conducted in Beijing on August 2015 and in Hainan on February 2016. The investigation of lesion length is carried out at 14 days after inoculation with Xoo strain PXO86. Rice varieties TP309, MH86, and D62B possess the homozygous *Xa5* gene. CK-TP is a $T_2$ plant from TP309 with pCAMBIA1300 plasmid;CK-1TP, CK-2TP, and CK-3TP are the $T_3$ plants of the CK-TP; 119 and 123 are knockout mutants of *Xa5* from TP309; 133, O23, and O32 are $T_2$ in-frame mutant plants, from TP309 and have the same mutation of the 32nd amino acid deletion in the Xa5 protein; 119-1, 119-2 and 119-3 are $T_3$ plants from 119; Similarly, 123-1, 123-2, 123-3, 123-4 are $T_3$ plants from 123; 133-1, 133-2, 133-3, 133-4 are $T_3$ plants from 133. CK-1MH and CK-2MH are homozygous $T_3$ plants from MH86; 23-1, 278-1, and 278-2 are $T_3$ knockout mutants in MH86 background; 3-1, 3-2, 3-3, 3-4, 3-5 are homozygous $T_3$ plants in MH86 background, they are all have the same mutation with 133, O23, and O32; 217-1, 217-2, 217-3, 217-4, 217-5 are homozygous $T_3$ plants, they are lack 4 amino acids from the 29th to 32nd in Xa5 protein; 235-1 are homozygous $T_3$ plant, in which the amino acid sequence of Xa5 from the 32nd to 40th site is replaced by a different 5- amino acids sequence. CK-1D6 is a homozygous $T_3$ plant from D62B; 179-1 is a $T_3$ homozygous knockout plant in D62B background; 180-1 is a $T_3$ homozygous mutant in D62B background, which have three amino acids deletion from 32nd to 34th amino acid in the Xa5 protein; 260-1 is a $T_3$ homozygous mutant in D62B background that has an amino acid insertion between the 32nd and the 33th amino acid in the Xa5 protein. Bars represent the average ±SD of three biological repeats. Different letters above columns indicate significant differences at $P<0.05$ as determined by a one-way ANOVA followed by post hoc Tukey honest significant difference (HSD) analysis.

**Figure S3. Homology analysis between TFIIAγ1 and TFIIAγ5 (Xa5)**

The comparison of *TFIIAγ1* to *TFIIAγ5* (*Xa5*) gene in amino acid sequence (A) and cDNA sequence (B).

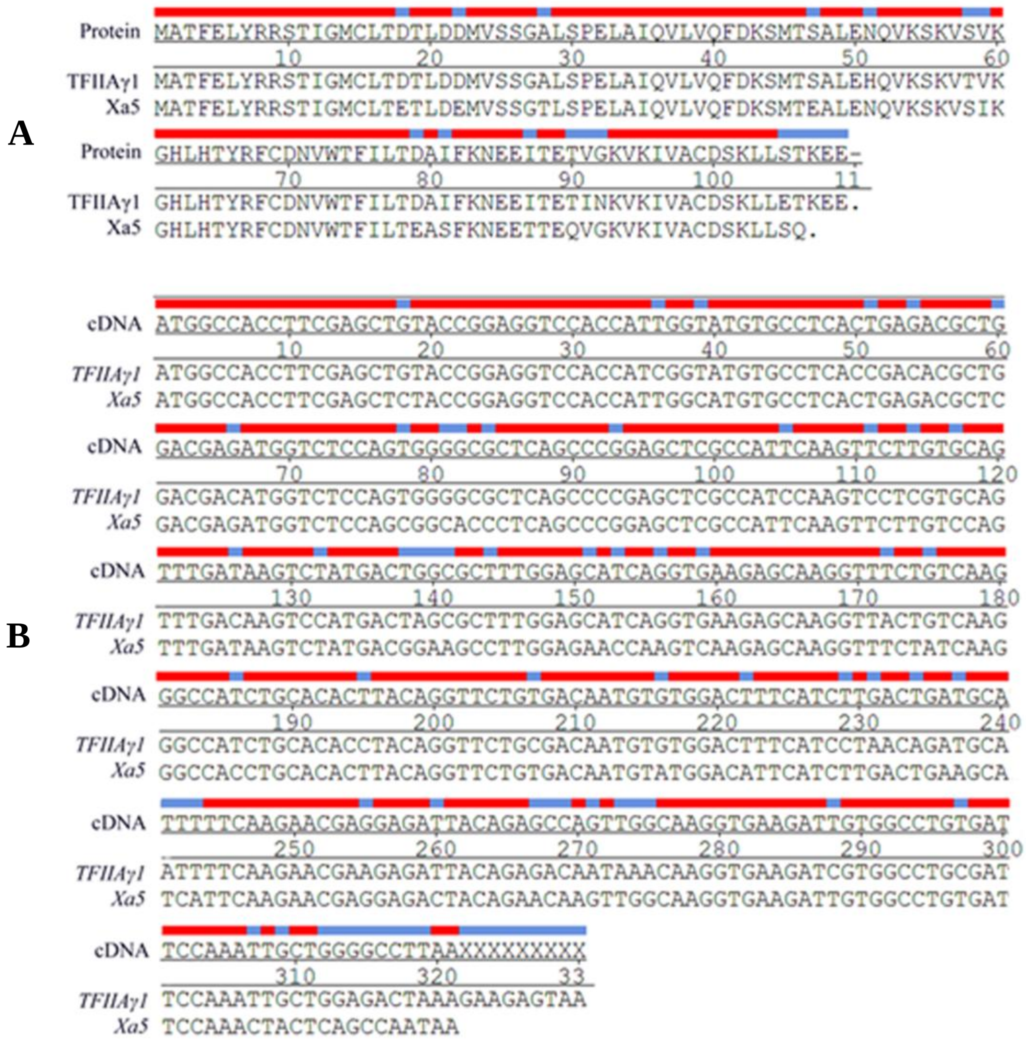

**Table S1. Primer sequences.**

| Oligonucleotide | Sequence (5'-3') |
|---|---|
| Xa5F | AGAGAAATCTGGCGTCTCGTC |
| Xa5R | TACGTGTCGGACGTGAATGG |
| TFⅡA-gamma-1F | GAGGGAGGGATGCGATACTTG |
| TFⅡA-gamma-1R | AGCCTATGAAGCAAGCAGGAA |
| Hpt-F | CGCCGATGGTTTCTACAA |
| Hpt-R | GGCGTCGGTTTCCACTAT |
| Fok-F | CTACAGGGGAAAGCACCTGG |
| Fok-R | ATAGGCAGATTGTAGCCGCC |

**Table S2.The mutation types of the gene *TFIIAγ1*.**

| | | |
|---|---|---|
| ***TFIIAγ1*** | **T**GGTCTCCAGTGGGGCGCTCAGCCCCGAGCTCGCCATCCAAGTCCTCGTGC**A** | |
| **148T0-1** | **T**GGTCTCCAGTGGGGCGCTCAGCCC ———TCGCCATCCAAGTCCTCGTGC**A** | **-5** |
| **148T0-2** | **T**GGTCTCCAGTGGGGCGCTCAGCCC——GCTCGCCATCCAAGTCCTCGTGC**A** | **-3** |
| **148T0-3** | **T**GGTCTCCAGTGGGGCGCTCAGCC—————————————CCTCGTGC**A** | **-19** |
| ***TFIIAγ1*** | **T**GGTCTCCAGTGGGGCGCTCAGCCCCGAGCTCGCCATCCAAGTCCTCGTGC**A** | |
| **207T0-1** | **T**GGTCTCCAGTGGGGCGCTCAGCCCC———TCGCCATCCAAGTCCTCGTGC**A** | **-4** |
| **207T0-2** | **T**GGTCTCCAGTGGGGCGCTCAGCCCC ——— CGCCATCCAAGTCCTCGTGC**A** | **-5** |
| **207T0-3** | **T**GGTCTCCAGTGGGGCGCTCAGCCCC ——CTCGCCATCCAAGTCCTCGTGC**A** | **-3** |
| ***TFIIAγ1*** | **T**GGTCTCCAGTGGGGCGCTCAGCCCCGAGCTCGCCATCCAAGTCCTCGTGC**A** | |
| **221T0-1** | **T**GGTCTCCAGTGGGGCGCTCAGCCCC———TCGCCATCCAAGTCCTCGTGC**A** | **-4** |
| **221T0-2** | **T**GGTCTCCAGTGGGGCGCTCAGCCCC — GCTCGCCATCCAAGTCCTCGTGC**A** | **-2** |
| ***TFIIAγ1*** | **T**GGTCTCCAGTGGGGCGCTCAGCCCCGAGCTCGCCATCCAAGTCCTCGTGC**A** | |
| **237T0-1** | **T**GGTCTCCAGTGGGGCGCT———————CTCGCCATCCAAGTCCTCGTGC**A** | **-10** |
| **237T0-2** | **T**GGTCTCCAGTGGGGCGCTCAGCCC————GCCATCCAAGTCCTCGTGC**A** | **-7** |
| **237T0-3** | **T**GGTCTCCAGTGGGGCGCTCAGCCC———CTCGCCATCCAAGTCCTCGTGC**A** | **-4** |
| **237T0-4** | **T**GGTCTCCAGTGGGGCGCTCAGCCCC ——CTCGCCATCCAAGTCCTCGTGC**A** | **-3** |
